# In Vitro Assessment of a Doubly Adjuvanted Self-Emulsified Nanoemulsion as a Delivery Vehicle for Antigenic Proteins

**DOI:** 10.3390/pharmaceutics17070870

**Published:** 2025-07-02

**Authors:** Evgenia Tsanaktsidou, Maritsa Margaroni, Evdokia Karagouni, Costas Kiparissides, Olga Kammona

**Affiliations:** 1 Chemical Process and Energy Resources Institute, Centre for Research and Technology Hellas, P.O. Box 60361, 57001 Thessaloniki, Greece; jtsanaktsidou@certh.gr (E.T.); costas.kiparissides@certh.gr (C.K.); 2 Immunology of Infection Laboratory, Hellenic Pasteur Institute, 11521 Athens, Greece; mmargaroni@pasteur.gr (M.M.); ekaragouni@pasteur.gr (E.K.); 3 Department of Chemical Engineering, Aristotle University of Thessaloniki, 54124 Thessaloniki, Greece

**Keywords:** self-nanoemulsifying drug delivery systems, antigenic proteins, nanoemulsion, protein release, adjuvant, vaccine carrier

## Abstract

**Background/Objectives:** Leishmaniasis is a prevailing infectious disease transmitted via infected phlebotomine sandflies. The lack of an efficient vaccine with respect to immunogenic antigens and adjuvanted delivery systems impedes its control. Following the induction of immune responses in mice vaccinated with multi-epitope *Leishmania* peptides (LeishPts) encapsulated in doubly adjuvanted self-nanoemulsifying drug delivery systems (ST-SNEDDSs), this study aims to assess ST-SNEDDS-based nanoemulsions as vehicles for the delivery of antigenic proteins. **Methods:** Model antigens (e.g., BSA-FITC, OVA) were encapsulated in ST-SNEDDS after being complexed with the cationic phospholipid dimyristoyl phosphatidylglycerol (DMPG) via hydrophobic ion pairing. The nanoemulsions were characterized with respect to droplet diameter, zeta potential, stability, protein loading, protein release from the nanodroplets in different release media and cell uptake. **Results:** Both model antigens exhibited high encapsulation efficiency (>95%) and their release from the nanodroplets was shown to be strongly affected by the type of release medium (e.g., PBS, FBS 10% *v*/*v*) and the ratio of its volume to that of the oily phase, in agreement with predictions of protein release. Protein-loaded nanoemulsion droplets labeled with Cy-5 were found to be efficiently taken up by macrophages (J774A.1) in vitro. However, no colocalization of the labeled nanodroplets and BSA-FITC could be observed. **Conclusions:** It was revealed that in contrast with LeishPts, whole protein molecules may not be appropriate antigenic cargo for ST-SNEDDS formulations due to the rapid protein release from the nanodroplets in release media simulating in vitro culture and in vivo conditions such as FBS 10% *v*/*v*.

## 1. Introduction

Vaccination can be viewed as a key strategy to control infectious diseases such as polio, smallpox, measles, diphtheria, meningitis A, tetanus, etc. In this regard, great effort has been put into the development of candidate vaccines for leishmaniasis, based on *Leishmania* parasites, recombinant *Leishmania* proteins, chimeric peptides, DNA, etc. [1,2,3,4]. Nevertheless, despite these intensive efforts, there exists no approved vaccine against *Leishmania* for human use [5,6]. Regarding canine leishmaniasis, only LetiFend^®^ (LETI Pharma, S.L.U., Madrid, Spain) out of the four vaccines that had been licensed in Europe (CaniLeish^®^ (VIRBAC, Carros, France) and LetiFend^®^) and Brazil (Leishmune^®^ (Zoetis Indústria de Produtos Veterinários LTDA, Campinas SP, Brazil) and Leish-Tec^®^ (Ceva Saúde Animal LTDA, Paulinia SP, Brazil)) is still authorized [7,8,9,10]. LetiFend^®^ contains the Recombinant Protein Q from *Leishmania infantum* and is sold as a lyophilized powder and water for injection that can be mixed to form an injectable solution. Letifend^®^ contains no additional adjuvant [7,11,12].

The lack of an effective vaccine for leishmaniasis could potentially be due to the absence of appropriate delivery systems and/or adjuvants [2] as well as the nature of the proposed antigen (i.e., antigens with high molecular weight and/or increased T cell epitope density are considered more immunogenic) [13].

Vaccine adjuvants comprise various synthetic or naturally occurring materials which boost the antigen’s immunological effect. Among them, oil in water (o/w) emulsions based on squalene (e.g., MF59) have been shown to enhance the immune responses to influenza vaccine antigens [14,15], whereas the additional presence of a-tocopherol in the o/w emulsion (e.g., AS03) has been revealed to further enhance the immune responses [16,17,18]. In addition, Toll-like receptor (TLR) agonists as immunostimulants have been found to significantly increase the immune responses against the administered antigen and dramatically enhance antigen cross-presentation by antigen-presenting cells such as dendritic cells (DCs) [13].

Lipid- and polymer-based nanocarriers have recently gained a lot of attention concerning the development of vaccine formulations against *Leishmania*, as they are vehicles for antigen and/or adjuvant delivery, while at the same time the majority of them can independently act as adjuvants. Among the lipid nanocarriers, liposomes, lipoplexes and solid lipid nanoparticles (SLNs) have been widely used [19].

Self-nanoemulsifying drug delivery systems (SNEDDS) are isotropic mixtures of oil, surfactant and cosurfactant freely forming oil-in-water (o/w) nanoemulsions upon mixing with an aqueous phase [20]. Emulsification occurs when the change in entropy which favors dispersion surpasses the necessary energy to increase the surface area of the dispersion [21,22]. The formed nanoemulsions are thermodynamically stable and are characterized by extremely small droplet diameters (i.e., <50 nm) [20]. The development of successful SNEDDS formulations is strongly dependent on the physicochemical characteristics and weight ratios of the excipients (e.g., oils, surfactants, cosurfactants) and the drug properties (e.g., lipophilicity/hydrophilicity, polarity, pKa) which influence the self-emulsification process. Oils (e.g., medium- and long-chain triglycerides, propylene fatty acid esters, etc.) are probably the most important excipients which govern the impulsiveness of self-nanoemulsification, the solubility of the drug and the size of the nanoemulsion droplets. A mixture of oils can be used to meet the requirements for drug solubilization and nanoemulsion formation. Surfactants (e.g., polyoxyethylene castor oil, polyoxyethylene stearate, polysorbates, etc.) facilitate the dispersion process by decreasing the interfacial tension. Various surfactants can be used for the development of SNEDDS, either alone or in combination. In the latter case, at least one surfactant should exhibit high hydrophilic–lipophilic balance (HLB). Co-surfactants with high HLB values can also be used to further decrease the nanodroplet size. Following the selection of the excipients, a factorial design is applied to identify the excipients’ weight ratios, resulting in the instant formation of transparent/translucent nanoemulsions. These ratios define the self-nanoemulsifying region in a ternary phase diagram. A mixture of the excipients from this region is finally selected for the incorporation of drugs [21,23].

**Figure 1:**
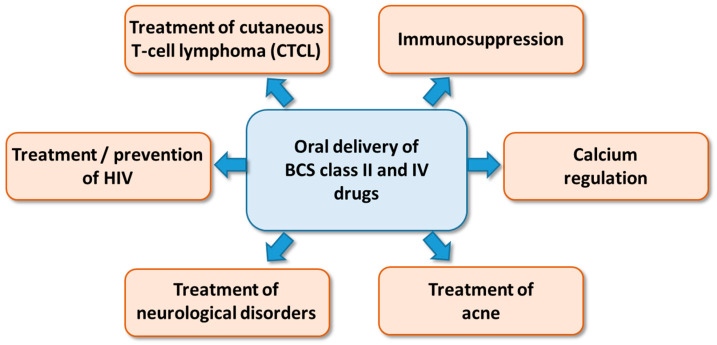
Applications of SNEDDS in the pharma industry [28].

Compared with other nanocarriers, SNEDDS can be effortlessly scaled up and have lower manufacturing costs [24]. They are usually selected for the enhancement of the oral bioavailability of hydrophobic drugs (e.g., cyclosporine A), for which there exist marketed formulations (Figure 1) such as Sandimmune^®^ and Neoral^®^ (Novartis, Basel, Switzerland) [20]. SNEDDS can be administered in the form of an oil phase (i.e., mixture of excipients) and are anticipated to convert to o/w nanoemulsions on the intestinal mucosa [25]. Lately, SNEDDS have been assessed for the delivery of hydrophilic biomolecules (e.g., therapeutic peptides) via the oral administration route [26,27,28,29]. An in vivo pharmacokinetic study of insulin glargine (IG) administered to rats both intravenously (i.v.) and orally (i) in the form of a solution and (ii) in the form of a IG-HIP (hydrophobic ion pair) complex encapsulated in SNEDDS revealed a maximum insulin concentration equal to 43.5 ± 5.4 mIU L^−1^ for the i.v. injection in comparison with 26.3 ± 8.1 mIU L^−1^ for SNEDDS and 4.3 ± 2.1 mIU L^−1^ for the oral IG solution 120 min after dosing. The orally administered IG-HIP complex in the SNEDDS formulation exhibited a higher bioavailability (e.g., 2.13%) in comparison with the IG solution (e.g., 0.27%) [29].

Additionally, self-emulsifying drug delivery systems (SEDDS) encapsulating the model antigen bovine serum albumin (BSA) and adjuvanted with Lipid A from Salmonella Minnesota R595 or squalene were evaluated in vivo for their efficiency. The developed SEDDS were found to induce both systemic and mucosal immunity after being orally administered to mice [24]. More recently, a stable (>20 weeks) oil-in-water (o/w) nanoemulsion (∅ < 30 nm), doubly adjuvanted with squalene and α-tocopherol and loaded with multi-epitope antigenic peptides, was developed based on SNEDDS technology as a potential vaccine formulation against *Leishmania*. The intramuscular (i.m.) administration of the nanoemulsion to a BALB/c model was shown to induce antigen-specific CD4+ memory T cells and IFNγ-producing CD4+ T cells, denoting its potential as a vaccine nanocarrier [27].

In the present study, the previously developed doubly adjuvanted o/w nanoemulsion [27] was loaded with model antigenic proteins (e.g., BSA, OVA) in order to be assessed regarding its potential to deliver large and consequently more immunogenic antigens such as the multiepitope chimeric protein LiChimera. The latter was revealed to induce both humoral and cellular immune responses [13] and to confer significant protection against *Leishmania* [30] following its encapsulation in cationic liposomes adjuvanted with imiquimod [13]. The formed nanoemulsions were characterized regarding their physicochemical properties, loading and release of the model antigens from the nanoemulsion droplets. In addition, the distribution coefficient LogD_o/w_ of the antigenic proteins between the nanoemulsion droplets (oil phase, o) and the release medium (aqueous medium, w) was determined in an attempt to theoretically predict their release profile from the nanoemulsion droplets [24,25]. Finally, the in vitro uptake and intracellular localization of the nanoemulsion in antigen-presenting cells (APCs) was examined.

## 2. Materials and Methods

### 2.1. Materials

Albumin-fluorescein isothiocyanate conjugate (BSA-FITC), albumin from chicken egg white (OVA), 1,2-Dimyristoyl-sn-glycero-3-phospho-rac-(1-glycerol) sodium salt (≥99%), macrogol (15)-hydroxystearate (Kolliphor^®^ HS15, Florham Park, NJ, USA), squalene (≥98%, liquid), (±)-α-Tocopherol (tested according to Ph. Eur.), N,N-dimethylformamide (DMF) (for molecular biology ≥99%) and phosphate-buffered saline (PBS, 10x, pH 7.4) were purchased from Sigma. Cyanine-5 carboxylic acid (Cy-5) was purchased from Lumiprobe Corporation (Westminster, MD, USA). Free samples of polyoxyethylated oleic glycerides (Labrafil M1944CS^®^) were kindly provided by Gattefossé (Saint-Priest, France). All other reagents were of analytical grade and commercially available.

### 2.2. Synthesis and Characterization of Protein/DMPG Complexes

BSA-FITC/DMPG and OVA/DMPG complexes were formed via hydrophobic ion pairing [26,27,28,29,31] so as to increase the protein (e.g., BSA-FITC, OVA) solubility in doubly adjuvanted SNEDDSs (ST-SNEDDSs) [31]. Considering the 103 cationic groups of BSA (e.g., 25 Arg, 17 His, 60 Lys and N-terminal site [24]), the 43 cationic groups of OVA (e.g., 15 Arg, 7 His, 20 Lys and N-terminal site [32]) and the anionic group of DMPG, protein/DMPG molar ratios equal to 1:103 and 1:43 were chosen for the formation of the BSA/DMPG and OVA/DMPG complexes, respectively. In more detail, a solution of DMPG (2.4 mg/mL) in deionized water (DI pH 6.0–6.5) was added dropwise to the protein solutions (10 mg/mL) in acidified water (pH 1.9) under magnetic stirring. The protein solutions became instantly cloudy, denoting the formation of the complexes. The protein/DMPG complexes were subsequently recovered through centrifugation (18,000× *g* at 25 °C for 20 min) [24,26] and lyophilized.

Fourier-transform infrared (FTIR) spectroscopy (Frontier FTIR spectrometer, Perkin Elmer, Shelton, CT, USA) was used to observe the interaction of DMPG with BSA-FITC and OVA. IR spectra of BSA-FITC, OVA and DMPG as well as BSA-FITC/DMPG and OVA/DMPG complexes were collected from KBr pellets at 25 °C. The protein content in the respective complex was determined by an HPLC analysis of the free protein in the supernatant. The analysis was performed on an Agilent 1200 HPLC system equipped with a UV detector [31]. An analytical column Bio SEC-3, 150 mm × 4.6 mm, 3 μm (Agilent Technologies, Santa Clara, CA, USA) was employed with a mobile phase at gradient elution. The mobile phase consisted of 100% buffer (0.1 M sodium phosphate buffer, pH 7.0 [33]) and the duration of the elution was 10 min. The flow rate was 1 mL/min, the detection wavelength was 214 nm and the injection volume was 10 μL.

### 2.3. Preparation and Characterization of Protein/DMPG-Loaded ST-SNEDDSs

For the formation of ST-SNEDDSs, the selected excipients/adjuvants (i.e., mixture of Labrafil M 1944 CS, Kolliphor HS15, Squalene and α-Tocopherol) were mixed in an Eppendorf tube (Eppendorf, Hamburg, Germany) and their mixture was subjected to sonication (40% amplitude, 45 s) in an ice bath with the aid of a microtip sonicator (Vibra Cell VC-505, Sonics & Materials, Inc. Newtown, CT, USA) to achieve a homogeneous oil phase. For the formation of ST-SNEDDS labeled with Cy-5, 0.5 mg of Cy5 was dissolved in DMF (50 mg/mL) and added to 1000 mg (~1 mL) of the oil phase (i.e., mixture of Labrafil M 1944 CS, Kolliphor HS15, Squalene and α-Tocopherol) prior to sonication (40% amplitude, 45 s). For the development of protein/DMPG-loaded ST-SNEDDSs, a specific amount of protein/DMPG complex (containing 1.6 or 5.7 mg BSA-FITC and 2.0–3.0 mg OVA) was added to 1000 mg (~1 mL) of the oil phase (i.e., mixture of Labrafil M 1944 CS, Kolliphor HS15, Squalene and α-Tocopherol [31]). To achieve the dissolution of protein/DMPG complexes in ST-SNEDDSs, the samples were gently vortexed and subsequently sonicated at a 40% amplitude for 2 min in an ice bath by means of a microtip sonicator (Vibra Cell VC-505, Sonics & Materials, Inc. Newtown, CT, USA) [26]. In this way, transparent oily solutions were obtained, signifying that the complexes were soluble in ST-SNEDDSs (oil phase). Subsequently, 2 mL of PBS was added dropwise under magnetic stirring to 1000 mg (~1 mL) of the oil phase, resulting in the formation of translucent nanoemulsions [31].

The formed nanoemulsions were characterized regarding their average droplet diameter and droplet size distribution (DSD) as well as their zeta potential via photon correlation spectroscopy (PCS) and aqueous electrophoresis measurements, respectively (Nano ZS90, Malvern Panalytical, Malvern, Worcestershire, UK). The protein loading in the nanodroplets was determined by HPLC analysis (Agilent Technologies, Santa Clara, CA, USA) of the free protein in the supernatant [26] after the centrifugation of nanoemulsion samples (18000× *g* at 20 °C for 20 min) by means of centrifugation filters 100 kDa.

### 2.4. Theoretical Prediction of Protein Release from the Nanoemulsion Droplets

In order to predict the release profile of BSA-FITC and OVA from the nanoemulsion droplets in PBS (pH 7.4) and FBS 10% *v*/*v* (pH 7.8), the distribution coefficient LogD_o/w_ of the antigenic proteins (solutes) between the nanodroplets (oil phase, o) and the release medium (aqueous medium, w) was initially determined by the following equation [25].

LogD_o/w_ = [protein]_o_/[protein]_w_ (1)

where [protein]_o_ and [protein]_w_ denote the concentration (solubility) of the antigenic proteins in the oil phase and aqueous medium, respectively.

The solubility of the antigenic proteins in the oil phase was calculated from the measured protein loading, whereas their solubility in the aqueous media was experimentally measured as follows. A small amount (e.g., 2 mg) of protein/DMPG complexes was suspended in 0.1 mL of aqueous medium (PBS, FBS 10% *v*/*v*) in Eppendorf tubes. The suspensions were incubated at 37 °C under shaking at 250 rpm for 1 and 2 h. They were then centrifuged at 18,000× *g* for 20 min, and the protein (e.g., BSA-FITC, OVA) content in the supernatant was quantified by HPLC [24], as previously described.

Finally, the theoretical amount of protein released from the nanoemulsion droplets in PBS and FBS 10% *v*/*v* was also calculated via the following equation [24,25] for eight V_w_/V_o_ ratios (e.g., 10, 30, 100, 150, 300, 600, 1200 and 1500).

Protein released (%) = 100 − [100/(1 + (V_w_/V_o_ × D_o/w_))] (2)

where V_w_ is the volume of the release medium and V_o_ is the volume of the nanodroplets.

### 2.5. In Vitro Release Study

To verify the predictions, in vitro release studies of BSA-FITC and OVA from the nanoemulsion droplets were carried out in PBS and FBS 10% *v*/*v* for various V_w_/V_o_ ratios (e.g., 10, 100, 150 and 300). In brief, vials containing protein-loaded ST-SNEDDS dispersed in PBS or FBS 10% *v*/*v* were incubated in a thermomixer (Thermomixer Compact, Eppendorf, Hamburg, Germany) at 37 °C and 250 rpm. At pre-specified time points (e.g., 0, 0.25, 0.5, 1, 2, 4, 6, 8, 12 h), the samples were centrifuged at 18,000× *g* and 20 °C for 20 min using centrifugation filters with MWCO 100 kDa. The protein quantity in the aqueous phase (e.g., PBS and FBS 10% *v*/*v*) was determined by HPLC as previously described.

### 2.6. Nanoemulsion Stability

The storage stability of the formed nanoemulsions was studied in PBS at 4 °C. The nanoemulsions were incubated at 4 °C and their droplet size distribution (DSD) was measured at 0, 4 and 12 weeks. Furthermore, the stability of the nanodroplets was examined in PBS and FBS 10% *v*/*v* at 37 °C, at 0, 2, 4 and 12 h.

### 2.7. Uptake Studies

In order to perform uptake and confocal studies, J774A.1 cell line (ATCC number: TIB-67) derived from mouse was used. J774A.1 cells (1 × 10^6^/mL) were seeded in 24-well cell culture plates and were incubated for 2 h in 5% CO_2_ and at 37 °C to allow cell adherence. Subsequently, cells were exposed to 260 μg/mL of Cy5-labeled ST-SNEDDS-BSA-FITC for 2 h and 24 h and were analyzed using flow cytometry. For confocal studies, 4 × 10^5^ cells/mL were transferred on 11 mm glass slides, incubated for 2 h, and then stimulated with 260 µg/mL of Cy5-labeled ST-SNEDDS-BSA-FITC for 30 min on ice. Afterwards, cells were washed with warm complete RPMI medium in order to remove non-phagocytosed ST-SNEDDS-BSA-FITC and incubated for 30 min, 2 or 4 h. During the final 30 min of incubation, cells were treated with LysoTracker® (Thermo Scientific Rockford, IL, USA) Red DND-99 to stain acidic cellular compartments. Finally, cells were washed and fixation was performed with 4% (*w*/*v*) paraformaldehyde (PFA) in PBS for 30 min at room temperature. Hoechst dye (1 µg/mL, 5 min) was used for nuclei staining. Each culture was prepared in duplicate, followed by an examination of the slides under a confocal microscope (Leica TCS-SP; Leica Microsystems, Wetzlar, Germany). Leica Confocal Software (LAS X version 5.3.0) was used for the acquisition and processing of images.

## 3. Results and Discussion

### 3.1. Synthesis and Characterization of Protein/DMPG Complexes

**Figure 2:**
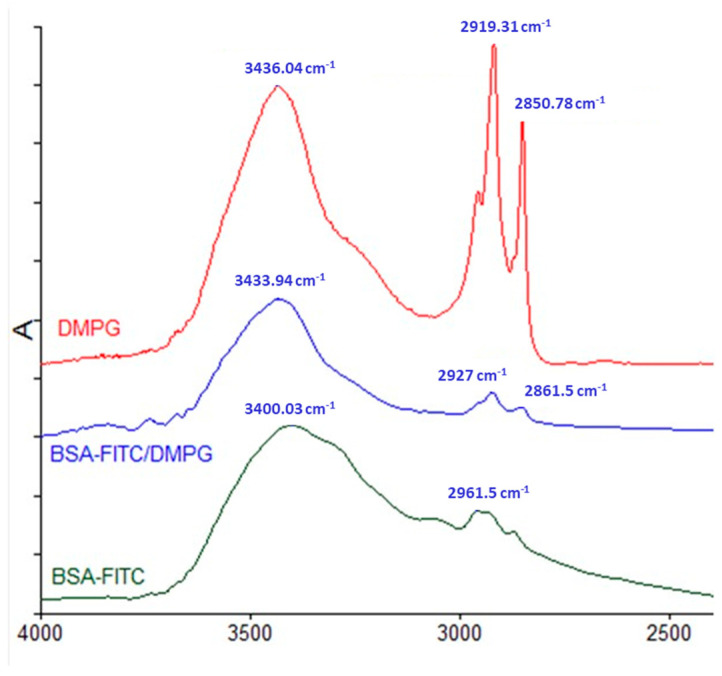
IR spectra of BSA-FITC, BSA-FITC/DMPG complex (BSA-FITC:DMPG molar ratio = 1:103) and DMPG.

Protein/DMPG complexes were successfully formed by the hydrophobic ion pairing method. FTIR spectroscopy (Frontier FTIR spectrometer, Perkin Elmer) was used to study the interaction of BSA-FITC and OVA with DMPG. Figure 2 shows the infrared spectra of DMPG, BSA-FITC/DMPG complex and BSA-FITC. As can be observed, the frequencies of the CH_2_ vibrations of DMPG (i.e., symmetric 2850.78 and antisymmetric 2919.31 cm^−1^ stretching) are shifted towards higher values in the BSA-FITC/DMPG spectrum (i.e., 2861.5 and 2927 cm^−1^, respectively). This could be attributed to the association of BSA-FITC with DMPG, resulting in lipid structural changes. The aforementioned vibrations are sensitive to lipid acyl chain conformation, whereas they are basically free of contribution from the protein component [34,35].

The protein content in the protein/DMPG complex as determined by HPLC was found to be equal to 46.4 ± 0.6% for BSA-FITC and 58.3 ± 0.5% for OVA. The complexation efficiency was shown to be high for both proteins (e.g., 96.3 ± 1.2% for BSA-FITC and 98.3 ± 0.2% for OVA), thus indicating that the proteins were efficiently complexed with the phospholipid.

### 3.2. Preparation and Characterization of Protein/DMPG-Loaded ST-SNEDDS

**Figure 3:**
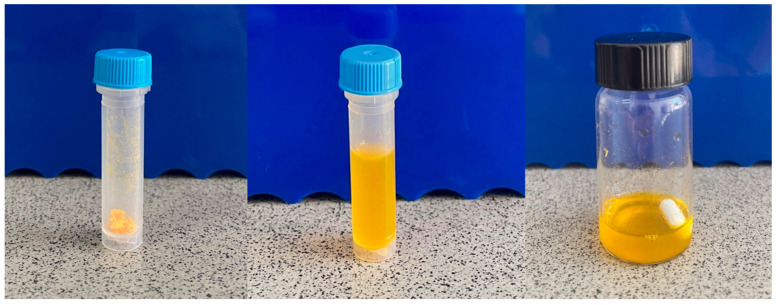
Lyophilized BSA-FITC/DMPG complex formed via hydrophobic ion pairing (**left**). ST-SNEDDS-BSA-FITC/DMPG, i.e., transparent oily solution consisting of a mixture of Labrafil M 1944 CS, Kolliphor HS15, Squalene and α-Tocopherol, where the BSA-FITC/DMPG complex has been dissolved with the aid of vortex and sonication at a 40% amplitude for 2 min in an ice bath with a microtip sonicator (**middle**). ST-SNEDDS-BSA-FITC/DMPG oil-in-water (o/w) nanoemulsion spontaneously formed by the dropwise addition of PBS to the oily solution ST-SNEDDS-BSA-FITC/DMPG under magnetic stirring (**right**).

All protein/DMPG complexes were shown to be efficiently dissolved in ST-SNEDDS. By adding PBS (pH 7.4) to the oil phase (ST-SNEDDS) containing the protein complexes, translucent nanoemulsions were freely formed (Figure 3). The characteristics of these nanoemulsions (potential vaccine formulations) are presented in Table 1. As can be seen, the formed nanoemulsions exhibited low droplet diameter (<30 nm), which could be attributed to the presence of Labrafil M1944CS, which has surfactant-like properties and therefore enhances the stabilization of the nanoemulsion droplets [26]. Regarding the droplet surface charges, blank nanoemulsions were characterized by neutral to positive zeta potential, which changed to negative with the incorporation of BSA-FITC and OVA. Finally, as can be observed in Table 1, increased encapsulation efficiency (≥95%) was achieved for both proteins.

### 3.3. Theoretical Prediction of Protein Release from the Nanoemulsion Droplets

**Figure 4:**
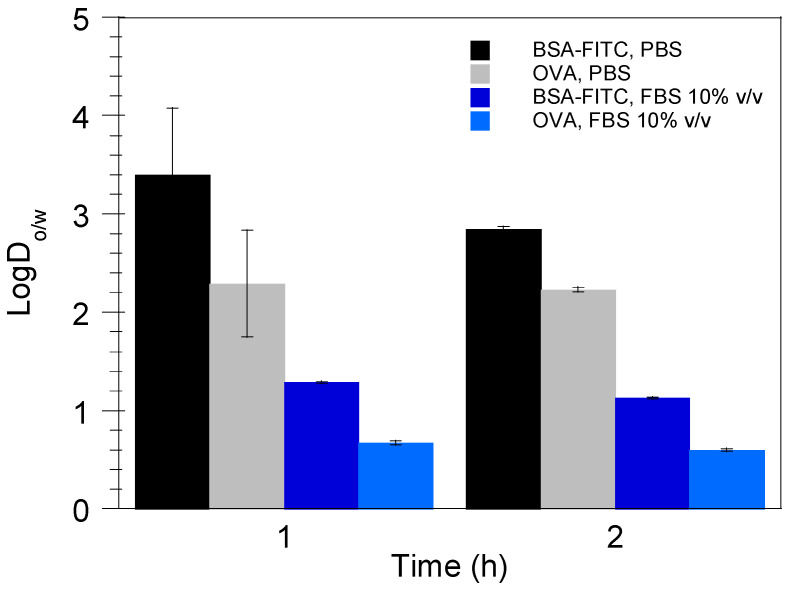
Distribution coefficient LogD_o/w_ of the antigenic proteins BSA-FITC and OVA (solutes) between the nanoemulsion droplets (oil phase, o) and the release media PBS and FBS 10% *v*/*v* (aqueous media, w) as determined by suspending the protein/DMPG complexes in PBS and FBS 10% *v*/*v*. Results are presented as mean ± SD (*n* = 3).

Figure 4 shows the distribution coefficient LogD_o/w_ of BSA-FITC and OVA between the nanodroplets (oil phase, o) and the release medium (aqueous medium, w) as determined by suspending the protein/DMPG complexes in PBS and FBS 10% *v*/*v* [24]. As can be observed, LogD_o/w_ values were higher for both antigenic proteins when the complexes were dispersed in PBS, indicating a slower protein release [25] in this medium in comparison with FBS 10% *v*/*v*. Additionally, for both release media, higher LogD_o/w_ values were observed for BSA-FITC, denoting a slower release rate from the nanoemulsion droplets as compared with OVA. It should be noted that the solubility of the protein/DMPG complexes in both release media slightly increased with time, as indicated by the decreased LogD_o/w_ values calculated after 2 h of incubation in PBS and FBS 10% *v*/*v* (Figure 4).

**Figure 5:**
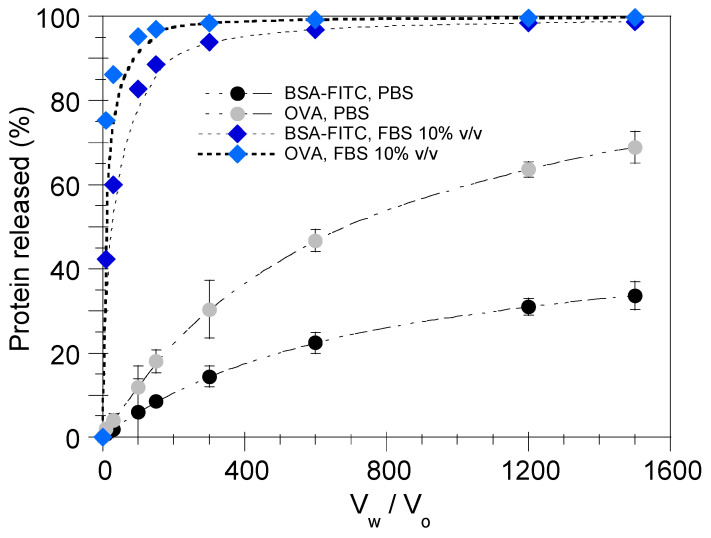
Effect of V_w_/V_o_ ratio on BSA-FITC and OVA release from the nanoemulsion droplets in PBS and FBS 10% *v*/*v*. Results are presented as mean ± SD (*n* = 3).

Figure 5 illustrates the effect of V_w_/V_o_ ratio on the percentage of BSA-FITC and OVA released in PBS, FBS 10% *v*/*v*. It is apparent that the amount of protein released from the nanoemulsion droplets increases with the increase in V_w_/V_o_ ratio, a phenomenon that is strongly enhanced when the encapsulated protein is OVA and the release medium is FBS 10% *v*/*v*. Notably, in the case of FBS 10% *v*/*v*, beyond a specific V_w_/V_o_ value (e.g., 300), the protein release reaches a plateau value and no longer increases, indicating an instant protein release from the nanoemulsion droplets for V_w_/V_o_ ≥ 300.

**Figure 6:**
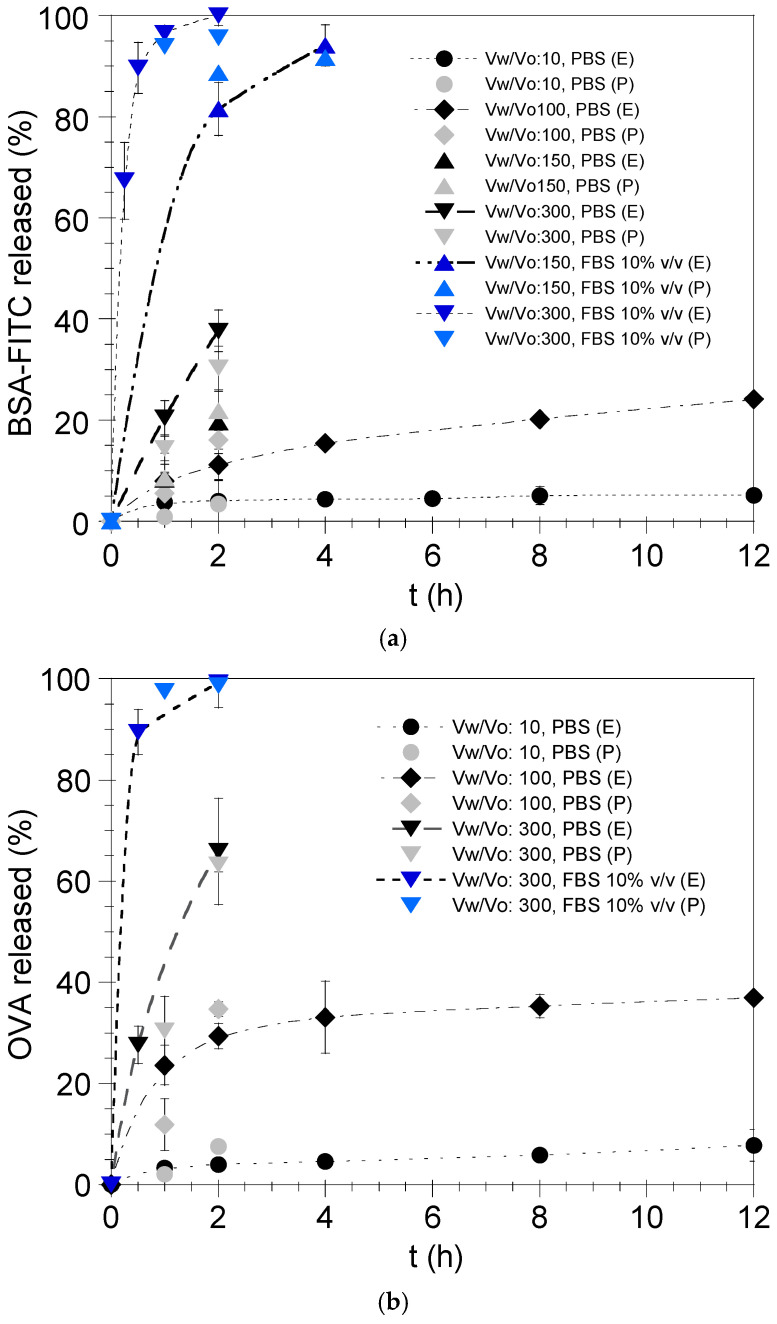
Experimental (E) and predicted (P) release of (**a**) BSA-FITC and (**b**) OVA from the nanoemulsion droplets in PBS and FBS 10% *v*/*v* (V_w_: volume of release medium and V_o_: volume of nanodroplets). Results are presented as mean ± SD (*n* = 3).

To verify the theoretical predictions, BSA-FITC and OVA release from the nanoemulsion droplets was examined in vitro in PBS at 37 °C as well as in FBS 10% *v*/*v* so as to simulate in vitro culture and in vivo conditions. Figure 6a,b depict the experimental results of BSA-FITC and OVA release, respectively, in both PBS and FBS 10% *v*/*v* at 37 °C for different V_w_/V_o_ ratios (e.g., 10, 100, 150, 300). As can be observed, the rate of protein release from the nanoemulsion droplets is dependent on the V_w_/V_o_ ratio. If the above ratio increases by decreasing V_w_/V_o_ and/or increasing V_w_, the percentage of protein which is released from the formulation increases dramatically. It is also apparent that the protein release rate from the nanoemulsion droplets depends on the release medium [24,25,36]. As can be seen in Figure 6a,b, for the same V_w_/V_o_ ratio (e.g., 300), the release of BSA-FITC and OVA is dramatically increased when the experiment is performed in FBS 10% *v*/*v*. This could be attributed to potential protein–protein interactions [37] between the protein molecules that are located at or have diffused to the droplet surface [25] and BSA molecules present in the release medium FBS 10% *v*/*v* (i.e., BSA is a principal component of FBS [38]). Additionally, by comparing Figure 6a,b, it can be observed that OVA exhibits a higher release rate in comparison with BSA-FITC independent of the V_w_/V_o_ ratio and the release medium (e.g., PBS, FBS 10% *v*/*v*). Taking into consideration that BSA and OVA have similar diffusion coefficients (10^−6^ cm^2^/s) in the nanodroplets as calculated based on the viscosity of the oil phase and the molar mass of the proteins [39], the time they need to diffuse to the surface of the nanoemulsion droplet (average diameter ≤ 30 nm) and consequently to the aqueous phase is minimal (<<1 s [25]). Accordingly, it can be inferred that the slower release of BSA-FITC from the oily nanodroplets in comparison with OVA is due to its lower LogD_o/w_ (Figure 4). The above-mentioned observations are in agreement with the predicted amount (%) of BSA-FITC and OVA released from the nanoemulsion droplets after 1 and 2 h in PBS and FBS 10% *v*/*v* for various V_w_/V_o_ ratios (Figure 6a and 6b, respectively), as well as with the results presented in Figure 5, showing the predicted percentage (%) of BSA-FITC and OVA released from the nanoemulsion droplets for different V_w_/V_o_ ratios.

**Figure 7:**
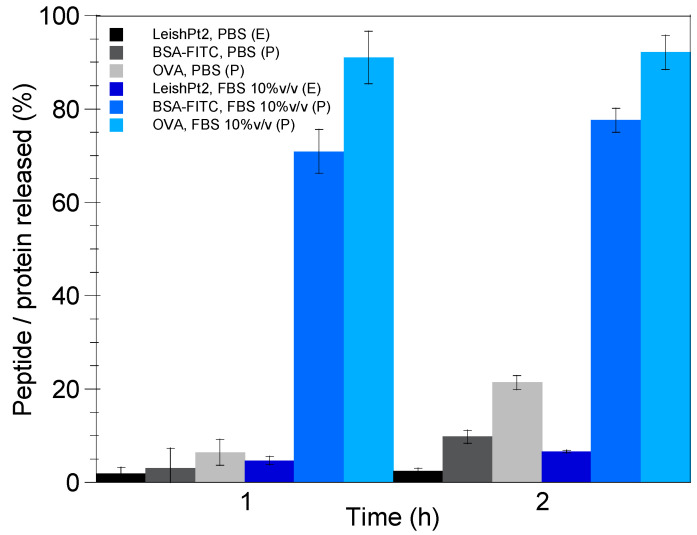
Experimental (E) and predicted (P) release of LeishPt2 and BSA-FITC and OVA, respectively, from the nanoemulsion droplets in PBS and FBS 10% *v*/*v* for V_w_/V_o_ ratio equal to 50. Results are presented as mean ± SD (*n* = 3).

It should be noted that a comparison between the protein (e.g., BSA-FITC, OVA) release results presented in Figure 6 and the previously published data on LeishPt2 (i.e., multi-epitope *Leishmania* peptide) released from the nanoemulsion droplets [31] reveals a slower peptide release in both release media (e.g., PBS, FBS 10% *v*/*v*), with the phenomenon being dramatically enhanced in the case of FBS 10% *v*/*v*. Taking into account that LeishPt2 release experiments were performed at a V_w_/V_o_ ratio equal to 50, and that the release profile is highly dependent on this ratio, Figure 7 attempts a direct comparison of the predicted amount (%) of BSA-FITC and OVA released from the nanoemulsion droplets after 1 and 2 h in PBS and FBS 10% *v*/*v* with the experimentally measured amount of LeishPt2 for a V_w_/V_o_ ratio equal to 50 [31].

It is apparent (Figure 7) that LeishPt2 exhibits a lower release rate in comparison with BSA-FITC and OVA, especially in FBS 10% *v*/*v*. Given that the diffusion coefficient of LeishPt2 in ST-SNEDDS is of the same order of magnitude (10^−6^ cm^2^/s) as those of BSA-FITC and OVA, as calculated based on the viscosity of the oil phase and the molar mass of the proteins [39], it can be suggested that the slower release of LeishPt2 from the oily nanodroplets in comparison with the antigenic proteins is due to its higher (>4) LogD _o/w_ (unpublished data). Finally, the enhancement of the difference between the peptide and the protein release rate in FBS 10% *v*/*v* could potentially be due to the protein–protein interactions [37] between BSA and OVA molecules located at or close to the nanodroplet surface [25] and BSA molecules present in FBS 10% *v*/*v*.

To identify the mechanism of protein release from the nanoemulsion droplets, the experimental release data were fitted to the Korsmeyer–Peppas model [40,41,42,43]. This model is represented by Equation (3).

M_t_/M_f_ = kt^n^ (3)

where M_t_/M_f_ is the fraction of the protein released from the nanodroplets at time t, k (time^−1^) is the release rate constant, which depends on the nanocarrier structure, and *n* is the diffusional exponent that is indicative of the release mechanism (e.g., Fickian diffusion: *n* < 0.45, non-Fickian transport: 0.45 < *n* < 0.89, Case II transport: *n* = 0.89, *n* > 0.89: super case II transport) [39,44,45].

It should be taken into account that the Korsmeyer–Peppas model can be only applied to experimental measurements corresponding to ≤60% of drug release [40,41,45].

Table 2 depicts the coefficients k and *n* of the Korsmeyer–Peppas model as calculated for protein release from the nanoemulsion droplets in PBS for two different V_w_/V_o_ ratios (e.g., 10 and 100). As can be seen in Table 2, the protein (e.g., BSA-FITC, OVA) release from the (labeled) nanodroplets is controlled by diffusion (Fickian diffusion), irrespective of the protein type or V_w_/V_o_ ratio [40,41,42,43,45]. This observation is in full agreement with Bernkop–Schnürch and Jalil [25], claiming that drug release from SNEDDS-based nanoemulsion droplets can be described as a simple diffusion process from an oily phase (nanodroplets) into an aqueous phase (release medium) in contrast with other types of nanocarriers (e.g., polymer nanoparticles), where other phenomena could also contribute to protein release [25].

### 3.4. Nanoemulsion Stability

**Figure 8:**
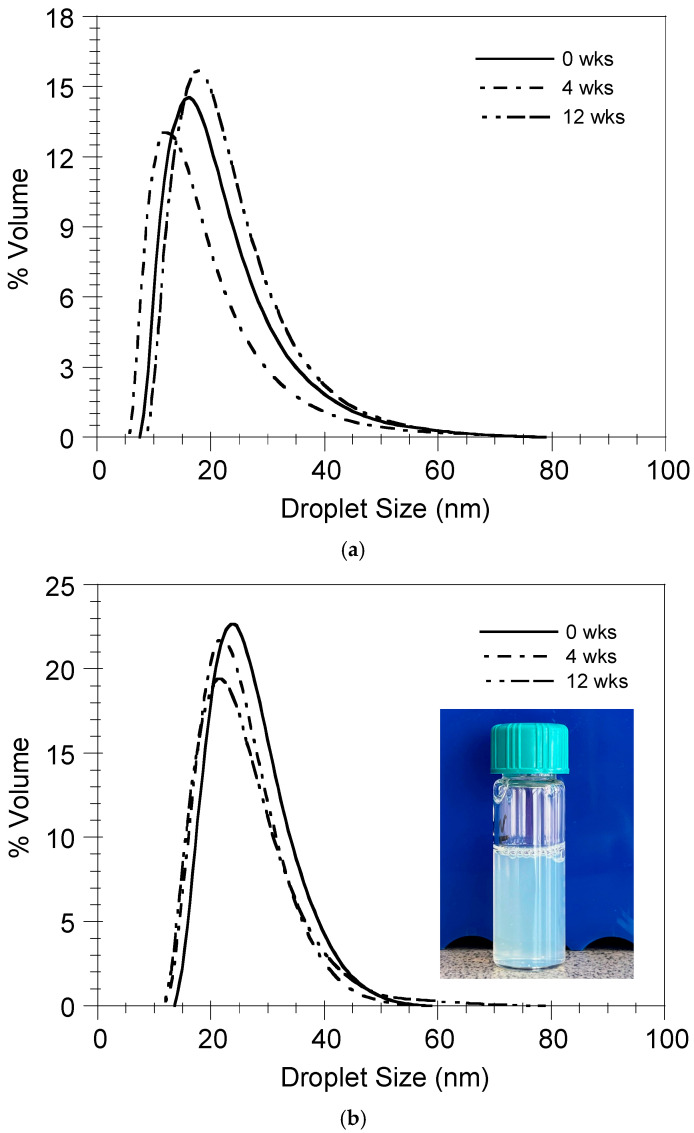
Storage stability of nanoemulsions in PBS at 4 °C. (**a**) ST-SNEDDS-BSA-FITC/DMPG-Cy5, (**b**) ST3-SNEDDS-OVA/DMPG (insert: nanoemulsion at 4 weeks).

Figure 8 shows the evolution of the droplet size distribution (DSD) of the nanoemulsions formed by the nanoemulsification of the BSA-FITC/DMPG- (Figure 8a) and OVA/DMPG-loaded (Figure 8b) ST-SNEDDS for numerous weeks at 4 °C. As can be observed in this Figure, independent of the protein loading, the nanoemulsions’ DSDs do not change dramatically with respect to time, denoting their stability in these conditions. A probable explanation could be the electrostatic repulsion between the nanodroplets exhibiting negative surface charges.

**Figure 9:**
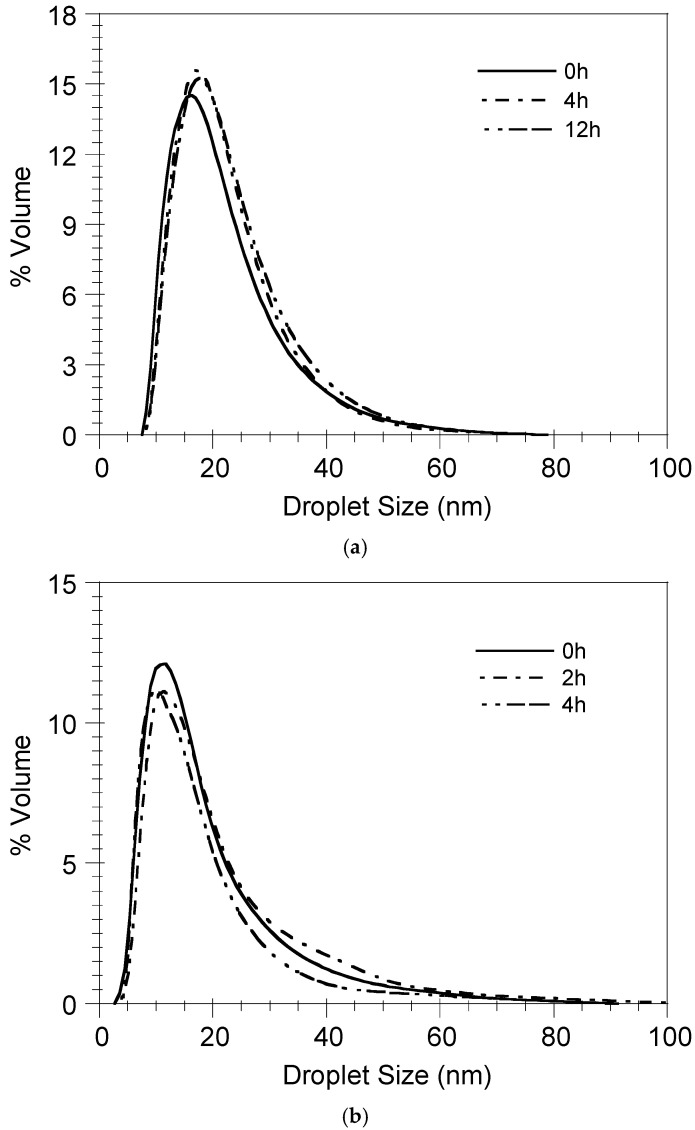
Stability of ST-SNEDDS-BSA-FITC/DMPG-Cy5 nanoemulsions in (**a**) PBS and (**b**) FBS 10% *v*/*v* at 37 °C.

Figure 9 depicts the evolution of the droplet size distribution of the ST-SNEDDS-BSA-FITC/DMPG-Cy5 nanoemulsion in PBS and FBS 10% *v*/*v* at 37 °C. As can be observed, the DSD does not change with time, indicating the stability of the nanoemulsion in both PBS and FBS 10% *v*/*v* at 37 °C and thus verifying diffusion as the release mechanism of BSA-FITC from the nanodroplets.

### 3.5. Uptake and Localization of ST-SNEDDS-BSA-FITC by Macrophages In Vitro

**Figure 10:**
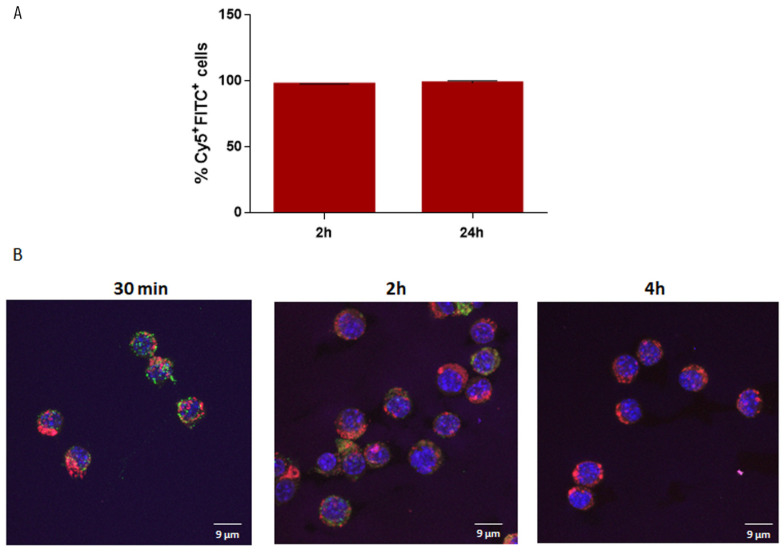
Uptake of ST-SNEDDS-BSA-FITC nanoemulsion droplets. J774A.1 cells were exposed to 260 µg/mL of Cy5-labeled ST-SNEDDS-BSA-FITC nanoemulsion for 2 h and 24 h at 37 °C, 5 % CO_2_. (**A**) The percentage (%) of Cy5+FITC+ cells as determined by flow cytometry. (**B**) Representative images showing the intracellular localization of Cy5-labeled ST-SNEDDS-BSA-FITC nanodroplets determined by confocal laser microscopy. Blue: nucleus; magenta: Cy5-labeled ST-SNEDDS; green: BSA-FITC; red: acidic compartments. Scale bar: 9 µm.

In order to investigate the ability of the Cy5-labeled ST-SNEDDS-BSA-FITC nanoformulation to be efficiently taken up by antigen-presenting cells, a prerequisite for immune response elicitation, J774 macrophage line cells were exposed in a concentration of 260 μg/mL of ST-SNEDDS-BSA-FITC for 2 h and 24 h. Flow cytometry revealed that from 2 h post exposure, 98 ± 0.2% of cells had taken up ST-SNEDDS-BSA-FITC, a percentage that remained stable after 24 h (98 ± 2%) (Figure 10a). The above results, in accordance with previous findings [31], indicate that the uptake process was completed during the first hours of exposure. This finding could be attributed to the size of the nanodroplets (<30 nm) [46], which enables cellular uptake via pino- or micropinocytosis, as well as their hydrophobic nature [47,48].

In order to further investigate the kinetics of the nanoformulation’s internalization in J774 cells, confocal studies were conducted. According to the obtained results, Cy5-labeled ST-SNEDDS-BSA-FITC nanoemulsion droplets were already internalized from 30 min and up to 2 h. After 4 h of incubation, Cy5-labeled nanodroplets were still located intracellularly; however, no FITC signal was detected, probably due to FITC catabolism. Interestingly, no colocalization of Cy-5 labeled nanodroplets and BSA-FITC was observed, neither at 30 min nor at 2 h, suggesting that BSA was released from the nanoemulsion droplets at early time points (Figure 10b). These findings imply that FITC-labeled BSA protein did not remain loaded in the nanodroplets, probably due to the increased release rate in FBS 10% *v*/*v*, as shown in Section 3.3. The above findings suggest that the ST-SNEDDS-based nanoemulsion droplets may be more appropriate carriers for molecules of lower molecular weight, such as peptides (e.g., multi-epitope *Leishmania* peptides) [31], instead of larger proteins like BSA or OVA and consequently LiChimera [13].

## 4. Conclusions

The model antigenic proteins BSA-FITC and OVA were successfully incorporated in doubly adjuvanted SNEDDS (ST-SNEDDS) in the form of protein/DMPG complexes. Increased encapsulation efficiencies were achieved (>95%). The controlled release of both model antigens was observed in PBS, in contrast to FBS 10% *v*/*v*, where rapid release was detected, in agreement with the calculated predictions of protein release for various V_w_/V_o_ ratios in the two release media. The formed nanoemulsions were found to exhibit enhanced storage stability (up to 12 weeks) in PBS at 4 °C. Additionally, the nanodroplet integrity was shown to be preserved in the release media at 37 °C (>12 h in PBS, up to 4 h in FBS 10% *v*/*v*). Finally, the nanoemulsion droplets were found to be efficiently taken up by macrophages in vitro, but no colocalization of Cy-5-labeled nanodroplets and BSA-FITC could be observed due to the rapid release of BSA from the nanoemulsion droplets in FBS 10% *v*/*v* at early time points, suggesting that, in contrast with multi-epitope peptides (e.g., LeishPt2), whole-protein molecules may not be appropriate antigenic cargo for ST-SNEDDS-based formulations. Still, future studies could be performed using antigenic proteins of different molecular weights and structures whose performance has been validated with other nanocarrier-based delivery systems.

## Figures and Tables

**Table 1:** Properties of nanoemulsions (potential vaccine formulations). Results are presented as mean ± SD (*n* = 3).

Nanoemulsion	Droplet Diameter (nm)	PDI	Zeta Potential (mV)	ST-SNEDDS Conc. in the Nanoemulsion (mg/mL)	Protein Loading in the oily Nanodroplets (wt%)	Protein Encapsulation Efficiency (%)	Cy5 Conc. in the Nanoemulsion (mg/mL)
ST-SNEDDS-BSA-FITC/DMPG	27.3 ± 1.2	0.07 ± 0.02	−3.5 ± 0.1	330	0.16 ± 0.02	95.3 ± 2.7	-
ST-SNEDDS-BSA-FITC/DMPG-Cy5	24.9 ± 0.2	0.21 ± 0.05	−3.8 ± 0.2	330	0.56 ± 0.00	98.4 ± 0.2	0.167
ST-SNEDDS-OVA/DMPG	27.3 ± 0.8	0.06 ± 0.03	−1.5 ± 1.1	330	0.25 ± 0.06	95.1 ± 1.8	-
ST-SNEDDS	28.6 ± 0.1	0.03 ± 0.02	1.8 ± 1.0	330	-	-	-
ST-SNEDDS-CY5	24.3 ± 0.8	0.23 ± 0.01	0.3 ± 1.0	330	-	-	0.167

**Table 2:** Coefficients of the Peppas–Ritger model for protein release from nanoemulsion droplets in PBS.

Formulation	V_w_/V_o_	Protein Loading (%*w*/*w*)	k	*n*	R^2^
ST3-BSA-FITC/DMPG	10	0.18	0.024	0.230	0.9630
ST3-BSA-FITC/DMPG-Cy5	10	0.56	0.009	0.304	0.9652
ST3-OVA/DMPG	10	0.29	0.030	0.369	0.9589
ST3-BSA-FITC/DMPG	100	0.16	0.082	0.403	0.9985

## Data Availability

The data presented in this study are available on request from the corresponding author.

## Abbreviations

The following abbreviations are used in this manuscript:

LeishPts	Multi-epitope *Leishmania* peptides
ST-SNEDDS	Doubly adjuvanted self-nanoemulsifying drug delivery systems
BSA-FITC	Bovine serum albumin–fluorescein isothiocyanate conjugate
OVA	Ovalbumin
DMPG	Dimyristoyl phosphatidylglycerol
PBS	Phosphate-buffered saline
FBS	Fetal bovine serum
DNA	Deoxyribonucleic acid
DC	Dendritic cell
SLNs	Solid lipid nanoparticles
SNEDDS	Self-nanoemulsifying drug delivery systems
SEDDS	Self-emulsifying drug delivery systems
IFNγ	Interferon gamma
LogD_o/w_	Distribution coefficient of the protein between the oil and aqueous phases
APC	Antigen presenting cell
Cy-5	Cyanine-5 carboxylic acid
FTIR	Fourier-transform infrared
KBr	Potassium bromide
HPLC	High-performance liquid chromatography
UV	Ultraviolet
DSD	Droplet size distribution
PCS	Photon correlation spectroscopy
MWCO	Molecular weight cut-off
LeishPt2	Multi-epitope *Leishmania* peptide
